# Editorial: Novel Applications of Chemometrics in Analytical Chemistry and Chemical Process Industry

**DOI:** 10.3389/fchem.2022.926309

**Published:** 2022-05-17

**Authors:** Alessandra Biancolillo, Angelo Antonio D’Archivio, Federico Marini, Raffaele Vitale

**Affiliations:** ^1^ Dipartimento di Scienze Fisiche e Chimiche, Università degli Studi dell’Aquila, Coppito, Italy; ^2^ Dipartimento di Chimica, Università degli Studi di Roma “La Sapienza”, Rome, Italy; ^3^ Univ. Lille, CNRS, LASIRE (UMR 8516), Laboratoire Avancé de Spectroscopie pour les Interactions, la Réactivité et l’Environnement, Lille, France

**Keywords:** chemometrics, multivariate statistics, high-dimensional data analysis, analytical chemistry, chemical process industry

Nowadays, thanks to many ground-breaking technological advances, old and new challenges in chemistry and chemical industry can be constantly addressed by means of cutting-edge analytical platforms, generating massive amounts of complex high-dimensional data. In this regard, chemometric approaches, enabling the extraction of the maximum content of meaningful information such data intrinsically encode, have been playing a key role. The present Research Topic collects a series of articles that actually corroborate this aspect, *i.e.*, how the utilisation of chemometrics could aid practitioners and operators in solving real-world issues in the two aforementioned domains, which, as for most scientific disciplines, are manifold and of rather diverse nature.

Several of these contributions have coped with fundamental methodological problems in the field of Multivariate Statistical Process Control (MSPC), that currently constitutes an undoubtedly *hot topic* given its inherent economic and social implications: Offermans et al. have proposed the use of conditional path modelling to infer the underlying intercorrelations linking different units of a production plant, Rocha de Olivera and De Juan have introduced the application of local Principal Component Analysis (PCA) for the assessment of non-synchronised batch process runs, Paris et al. have explored two different strategies for defining specification regions for raw industrial materals, while Strani et al. have fused near-infrared (NIR) and engineering sensors to construct MSPC control charts for polymerisation reaction monitoring.

Wide attention has also been paid to the world of food manufacturing and quality evaluation. In this sense, Ruiz et al. have developed a diagnostic tool resorting to the principles of Partial Least Squares regression (PLS) for compliant/defective product classification. Nieuwoudt et al. have exploited Analysis of variance-Simultaneous Component Analysis (ASCA) to determine the main sources of variation influencing the performance of various Fourier Transform-InfraRed (FTIR) spectrometers in a milk factory. Astolfi et al. have utilised dedicated chemometric techniques for the authentication of extra-virgin olive oil samples by Inductively Coupled Plasma-Mass Spectrometry (ICP-MS). Finally, Shao et al. have reviewed the state-of-the-art approaches for the electrochemical and biochemical sensor-based characterisation of tea specimens.

New light has also been shed on subjects apparently not yet well-established in the scientific community: Vitale et al., for instance, have addressed the problem of hyperspectral video processing through a hybrid modelling procedure encompassing spatial, spectral and temporal parametrisations of physico-chemical phenomena.

More theoretical aspects behind the use of chemometrics have been debated by Rutledge et al. who have compared several strategies for the estimation of the optimal complexity of multivariate statistical models.

Last but not least, Mancini and Rinnan as well as Alladio et al. have reported studies bridging elegantly the gap between theory and practice of multivariate statistics applications: the former have designed a solution for estimating waste wood heterogeneity coupling NIR spectroscopy, nested ANalysis Of VAriance (ANOVA) and PCA, the latter have devised a real-time predictive maintenance methodology (that combines Sparse Logistic PCA—SLPCA—and Soft Independent Modelling of Class Analogy—SIMCA) to prevent breakdowns during the evolution of automotive industrial processes.

Overall, as far as the editors are concerned, this Research Topic has surely permitted to stress the importance and relevance that data analysis and, more specifically, chemometrics can have in both basic and applied research scenarios.

